# The Tomato Transcription Factor SlNAC063 Is Required for Aluminum Tolerance by Regulating *SlAAE3-1* Expression

**DOI:** 10.3389/fpls.2022.826954

**Published:** 2022-03-15

**Authors:** Jian Feng Jin, Hui Hui Zhu, Qi Yu He, Peng Fei Li, Wei Fan, Ji Ming Xu, Jian Li Yang, Wei Wei Chen

**Affiliations:** ^1^State Key Laboratory of Plant Physiology and Biochemistry, College of Life Sciences, Zhejiang University, Hangzhou, China; ^2^State Key Laboratory of Conservation and Utilization of Bio-Resources in Yunnan, The Key Laboratory of Medicinal Plant Biology of Yunnan Province, National and Local Joint Engineering Research Center on Germplasm Innovation and Utilization of Chinese Medicinal Materials in Southwest China, Yunnan Agricultural University, Kunming, China; ^3^Research Centre for Plant RNA Signaling, Zhejiang Provincial Key Laboratory for Genetic Improvement and Quality Control of Medicinal Plants, College of Life and Environmental Sciences, Hangzhou Normal University, Hangzhou, China

**Keywords:** aluminum stress, expression regulation, metabolism, NAC transcription factor, oxalate

## Abstract

Aluminum (Al) toxicity constitutes one of the major limiting factors of plant growth and development on acid soils, which comprises approximately 50% of potentially arable lands worldwide. When suffering Al toxicity, plants reprogram the transcription of genes, which activates physiological and metabolic pathways to deal with the toxicity. Here, we report the role of a NAM, ATAF1, 2 and CUC2 (NAC) transcription factor (TF) in tomato Al tolerance. Among 53 NAC TFs in tomatoes, *SlNAC063* was most abundantly expressed in root apex and significantly induced by Al stress. Furthermore, the expression of *SlNAC063* was not induced by other metals. Meanwhile, the SlNAC063 protein was localized at the nucleus and has transcriptional activation potentials in yeast. By constructing CRISPR/Cas9 knockout mutants, we found that *slnac063* mutants displayed increased sensitivity to Al compared to wild-type plants. However, the mutants accumulated even less Al than wild-type (WT) plants, suggesting that internal tolerance mechanisms but not external exclusion mechanisms are implicated in SlNAC063-mediated Al tolerance in tomatoes. Further comparative RNA-sequencing analysis revealed that only 45 Al-responsive genes were positively regulated by SlNAC063, although the expression of thousands of genes (1,557 upregulated and 636 downregulated) was found to be affected in *slnac063* mutants in the absence of Al stress. The kyoto encyclopedia of genes and genomes (KEGG) pathway analysis revealed that SlNAC063-mediated Al-responsive genes were enriched in “phenylpropanoid metabolism,” “fatty acid metabolism,” and “dicarboxylate metabolism,” indicating that SlNAC063 regulates metabolisms in response to Al stress. Quantitative real-time (RT)-PCR analysis showed that the expression of *SlAAE3-1* was repressed by SlNAC063 in the absence of Al. However, the expression of *SlAAE3-1* was dependent on SlNAC063 in the presence of Al stress. Taken together, our results demonstrate that a NAC TF SlNAC063 is involved in tomato Al tolerance by regulating the expression of genes involved in metabolism, and SlNAC063 is required for Al-induced expression of *SlAAE3-1*.

## Introduction

Aluminum (Al) is the most abundant metal element in the earth’s crust and its solubility is determined by soil pH. Under acidic conditions, ionic Al is released into the soil solution, which is highly toxic to plant roots (Kochian, 1995). Therefore, Al toxicity in acidic soils constitutes one of the serious problems in the agricultural ecosystem. To cope with Al toxicity, plants have evolved sophisticated mechanisms at multiple levels (Kochian et al., 2015). Upon receiving Al toxic signal, a complicated signaling network might be triggered, which reprograms the expression of an array of Al-responsive genes (Liu et al., 2014). The expression reprogramming of these genes ultimately activates various physiological, biochemical, and metabolic processes. Based on the spatial location of these triggered processes, the implicated mechanisms could be assigned into either external exclusion mechanism or internal tolerance mechanism (Kochian et al., 2015). Among various external exclusion mechanisms, Al-induced organic acid anions secretion has been well-documented as a very important mechanism and widely adopted by many plants to chelate Al, forming non-toxic complexes (Yang et al., 2019; Chen et al., 2022). On the other hand, internal tolerance mechanisms have been related to the complexation of Al and subsequent compartmentation once Al has entered into cells (Ma et al., 2001).

Recent evidence showed that metabolic change plays an important role for cells to adapt to Al stress. Genome-wide transcriptional profiling revealed that a substantial amount of differentially expressed genes in response to Al stress are associated with metabolisms (Fan et al., 2014; Xu et al., 2017; Gao et al., 2020). In rice bean (*Vigna umbellata*), Al-induced formate accumulation is responsible for Al toxicity, and *VuFDH* is a gene encoding formate dehydrogenase that catalyzes formate degradation, thereby conferring Al tolerance (Lou et al., 2016a). Furthermore, rice bean *VuAAE3*, a gene coding for oxalyl-CoA synthetase has been functionally characterized to be involved in Al tolerance (Lou et al., 2016b). It seems that Al-induced accumulation of oxalate in the cytoplasm is detrimental to cells, and VuAAE3 is required for specifically degrading of oxalate to form oxalyl-CoA. AAE3 was first characterized in *Arabidopsis* (*Arabidopsis thaliana*) to be an oxalyl-CoA synthetase (Foster et al., 2012), and its functional homologs have been reported in *Saccharomyces cerevisiae* (Foster and Nakata, 2014), rice bean (Lou et al., 2016b), rice (*Oryza sativa*) (Peng et al., 2017), *Medicago truncatula* (Cheng et al., 2018), and wild soybean (*Glycine soja*) (Xian et al., 2020). Therefore, it appears that AAE3-dependent oxalate metabolism is a conserved mechanism to deal with both biotic and abiotic stresses (Chen et al., 2017). However, it remains unclear how the expression of *AAE3* genes was transcriptionally regulated in response to Al stress.

Transcription factors (TFs) are master regulators of transcription *via* interacting with special DNA sequences, namely, *cis* elements. A Cys2His2-type zinc finger TF, sensitive to proton rhizotoxicity 1 (STOP1), has been isolated by screening Arabidopsis mutants hypersensitive to low pH stress (Iuchi et al., 2007). STOP1 regulates not only the expression of genes associated with low pH tolerance but also an Al-activated malate transporter 1 (AtALMT1) gene to confer Al tolerance in Arabidopsis (Sawaki et al., 2009). To date, genes homologous to STOP1 have been characterized from a number of plant species, including wheat (*Triticum aestivum*) (Garcia-Oliveira et al., 2013), tobacco (*Nicotiana tabacum*), *Populus nigra*, *Lotus japonicas*, *Physcomitrella patens* (Ohyama et al., 2013), *Eucalyptus* (Sawaki et al., 2014), rice bean (Fan et al., 2015), pigeon pea (*Cajanus cajan*) (Daspute et al., 2018), tea (*Camellia sinensis*) (Zhao et al., 2018), soybean (*Glycine max*) (Wu et al., 2018), sweet sorghum (*Sorghum bicolor*) (Huang et al., 2018), and cotton (*Gossypium hirsutum*) (Kundu et al., 2019). Furthermore, several TFs belonging to WRKY and calmodulin-binding transcription activator family have been characterized to be involved in Al resistance by regulating the expression of genes related to organic acid anions secretion (Ding et al., 2013; Tokizawa et al., 2015; Li et al., 2018; Zhu et al., 2021). However, there is no evidence that these TFs are implicated in oxalate metabolism *via* regulation of the expression of *AAE3* genes.

The NAC [No apical meristem (NAM), Arabidopsis transcription activator 1/2 (ATAF1/2), and Cup-shaped cotyledon (CUC2)] proteins constitute a plant-specific TF family that are characterized with a conserved NAC domain at their N-terminals and a highly variable C-terminus (Olsen et al., 2005). In Arabidopsis and rice, a large number of NAC proteins have been reported to be involved in response to plant biotic and abiotic stresses (Olsen et al., 2005). Recently, several lines of evidence showed that NAC TFs could have been implicated in Al stress response in plants. First, Al stress could regulate the expression of *NAC* genes in rice (Moreno-Alvarado et al., 2017), tomato (Jin et al., 2020), and rice bean (Fan et al., 2014). Second, ectopic expression of a rice bean *NAC* gene, *VuNAR1* in Arabidopsis confers transgenic plants improved Al tolerance (Lou et al., 2020). Finally, it has been demonstrated that the SUPPRESSOR OF GAMMA RESPONSE 1 (SOG1) is implicated in responding to Al-induced DNA damage in Arabidopsis (Sjogren et al., 2015). Therefore, NAC TFs might constitute different layers of responses to protect plants from Al injury.

We have previously demonstrated that there are 7 out of 93 tomato *SlNAC* genes regulated by Al stress. Among them, *SlNAC063* was found to be most significantly upregulated in tomato root apex by Al (Jin et al., 2020). In this study, we functionally characterized *SlNAC063* in terms of Al stress by constructing CRISPR/Cas9 knockout (KO) transgenic lines. We further demonstrated that SlNAC063 is a master regulator of *SlAAE3-2* transcription, but its regulation on *SlAAE3-1* transcription is affected by Al stress.

## Materials and Methods

### Plant Materials and Growth Conditions

Seeds of tomato (*Solanum lycopersicum*) cultivar Ailsa Craig (AC) were obtained from Horticulture Research International (Warwick, United Kingdom). The *slnac063* mutants used in this study were constructed *via* CRISPR/Cas9 technology as described below. Seeds were sterilized with 10% NaClO (v/v) for 15 min, then washed with sterilized water five times to remove the residual NaClO. Seeds were soaked in sterilized water overnight and then sown on agar plates containing 1/5 strength Hoagland nutrient solution (pH 5.5) consisting of KNO_3_ (1.0 mM), Ca (NO_3_)_2_ (1.0 mM), MgSO_4_ (0.4 mM) and NH_4_H_2_PO_4_ (0.2 mM), and the micronutrients NaFeEDTA (20 μM), H_3_BO_3_ (3.0 μM), MnCl_2_ (0.5 μM), CuSO_4_ (0.2 μM), ZnSO_4_ (0.4 μM) and (NH_4_)_6_Mo_7_O_24_ (1 μM), with 0.8% Agar (Sigma-Aldrich, United States). Plates were kept in the dark at 4°C for 2 days and then seeds were germinated in a plant growth room with a daytime 16 h/24°C and 8 h/22°C night regime. After germination, uniform seedlings with primary root length about 3–4 cm were transferred to the 1/5 Hoagland nutrient solution (pH 5.0) with NH_4_H_2_PO_4_ concentration decreased to 10 μM for the following treatments.

### Expression Pattern Analysis

For the tissue-specific expression, seedlings (cv. AC) were exposed to 0 or 5 μM Al for 6 h. For the time-course experiment, seedlings were subjected to 5 μM Al for 0, 0.5, 1, 3, or 6 h. For the dose-response experiment, seedlings were subjected to 0, 5, 10, or 20 μM of AlCl_3_ for 6 h. For other treatments, the seedlings were exposed to a nutrient solution containing 5 μM AlCl_3_, 5 μM LaCl_3_, 1 μM CuCl_2_, or 5 μM CdCl_2_ for 6 h. All the samples were collected after various treatments and stored at −80°C until use.

### Quantitative Real-Time-PCR Analysis

Ribonucleic acid extraction of all samples was performed using the RNAprep pure Plant Kit (TIANGEN, China). First-strand cDNA was synthesized from 1 μg of total RNA using the PrimeScript™ RT Master Mix Kit (Takara, Japan). Quantitative RT-PCR was carried out on a LightCycler 480 RT-PCR system (Roche, Switzerland) using SYBR Green chemistry (Toyobo, Japan). Expression data of genes were normalized with the expression of tomato GAPDH (Cho et al., 2019). The primers are given in Supplementary Table 1.

### Cloning and Bioinformatics Analysis of SlNAC063

The 1069-bp coding sequence of SlNAC063 was amplified with primers of SlNAC063-green fluorescent protein (GFP)-F and SlNAC063-GFP-R (Supplementary Table 1) designed based on the tomato genome database from Phytozome v13^1^ (Goodstein et al., 2012). Multiple sequence alignment was performed using ClustalW program in the MEGA 7.0^2^ with default parameters (Gap opening penalty: 10.0; gap extension penalty: 0.1; protein weight matrix: Gonnet; residue-specific penalties: ON; hydrophilic penalties: ON; gap separation matrix: 4; end gap separation: OFF; use native matrix: OFF; delay divergent cutoff: 30%) (Kumar S. et al., 2016). Putative phosphorylation sites of SlNAC063 protein were searched at the NetPhos 3.1 Server^3^ (Blom et al., 1999, 2004).

### Transactivation Activity Assays

For analysis of the transactivation activity, the coding sequence and the truncated sequences of SlNAC063 were amplified by PCR with different pairs of gene-specific primers (Supplementary Table 1) and cloned into pGBKT7 at *Nde*I and *Sal*I sites (Brachmann and Boeke, 1997; Louvet et al., 1997). Then, the recombinant plasmids and pGBKT7 empty vector were transformed into yeast strain AH109. The transformed yeasts were plated on SD/-Trp medium or SD/-Histidine (His) medium containing 4 mM 3-Amino-1,2,4-triazole (3-AT) and incubated for 72 h at 30°C. Transactivation activity was assessed according to the growth status and production of blue pigment after the addition of X-α-gal (5-bromo-4-chloro-3-indolyl-α-D-galactopyranoside) on SD/-His medium.

### Subcellular Localization Analysis

The 1,069-bp coding DNA sequence (CDS) of SlNAC063 without the stop codon was amplified by PCR from wild-type (WT) root cDNA with primers listed in Supplementary Table 1, and then cloned into the *Pro35S::GFP* binary overexpression vector to produce the fusion construct *Pro35S::SlNAC063:GFP* using ClonExpress II One Step Cloning Kit (Vazyme, China). Then *Pro35S::SlNAC063:GFP* and the control vector (*Pro35S::GFP*) were transformed into *Agrobacterium tumefaciens* strain GV3101 and injected into 4-week-old tobacco leaves. GFP signal was observed and captured using confocal laser scanning microscopy (LSM710, Germany) after 48 h of infiltration.

### Construction of Tomato Transgenic Lines

The construction of SlNAC063 CRISPR/Cas9 KO lines was carried out following Zhang et al. (2017). Briefly, to generate CRISPR/Cas9-directed SlNAC063-KO construct, pKO-SlNAC063 (Supplementary Figure 1A), two sgRNAs that specifically target the 3rd exon of the target gene *SlNAC063* were selected using CRISPR-P 2.0^4^ (Liu et al., 2017) to produce four primers (Supplementary Table 1), which were used to amplify PCR fragment DT1-sgRNA1-DT2-sgRNA2 and then cloned into the *Bbs*I site in the CRISPR/Cas9 binary vector pHEE401E. Then the fragment was ligated into the binary vector pHEE401E by the restriction-ligation system (Wang et al., 2015). Then, the recombinant plasmid *pHEE401E-2sgRNA-SlNAC063* was transformed into wild-type tomato AC using the stable *A. tumefaciens*-mediated transformation method (McCormick et al., 1986; Gupta and Van Eck, 2016). The transgenic tomato lines were selected through their hygromycin resistance.

### Deoxyribonucleic Acid Extraction and Mutation Analysis

Total genomic DNA was extracted from tomato frozen leaves using a Plant Genomic DNA Kit (TIANGEN, China) and used as a template for amplifying the gene fragments, including target sites, using specific PCR primers. The PCR products were purified using TIANgel Midi Purification Kit (TIANGEN, China) and then sequenced to identify mutations. Primers used for this analysis are listed in Supplementary Table 1.

### Evaluation of Aluminum Tolerance

For Al tolerance evaluation, 1-week-old seedlings of WT plants and *slnac063* mutants were transferred to the 1/5 Hoagland nutrient solution containing 10 μM NH_4_H_2_PO_4_ (pH 5.0) either with or without 10 μM AlCl_3_ for 1 week. The treatment solution was renewed every other day. After treatment, biomass and the longest root length were measured. Seedlings were separated into roots and shoots and dried at 70°C for 48 h. After digestion with concentrated HNO_3_, Al content was determined by inductively coupled plasma mass spectrometry (ICP-MS) (Maher et al., 2001).

### Ribonucleic Acid-Sequencing and Bioinformatics Analysis

Total RNA was separately extracted from the roots of 4-day-old wild type and *slnac063* mutants, transferred to the 1/5 Hoagland nutrient solution with 10 μM NH_4_H_2_PO_4_ (pH 5.0) containing 0 or 10 μM AlCl_3_ for 7 days. The mRNA was enriched using oligo-dTs coupled with magnetic beads before being cut into 300 bp fragments. Then, RNA-Seq libraries were constructed and 150 base pairs pair-end sequencing was performed on an Illumina HiSeq platform. Gene expression abundance was represented by the reads per kilobase of transcript per million mapped reads (RPKM) value. Differentially expressed genes between WT and *slnac063* mutant (with or without AlCl_3_) were identified by DESeq2 Library (Anders and Huber, 2010). If | fold change| ≥ 2 and *P*-value < 0.05, genes were considered as differently expressed genes (DEGs). RNA-Seq data is available as accession number PRJNA785101 in the National Center for Biotechnology Information (NCBI) SRA database^5^.

### Statistical Analysis

Significance analysis of bioassays was performed using Microsoft Excel (version 2016, Microsoft Corporation, Redmond, WA, United States). Experimental data were analyzed with Student’s *t*-test or Tukey’s test at the level of *p* < 0.05. All the experiments were repeated independently three times.

## Results

### Characteristics of SlNAC063 Protein

**FIGURE 1:**
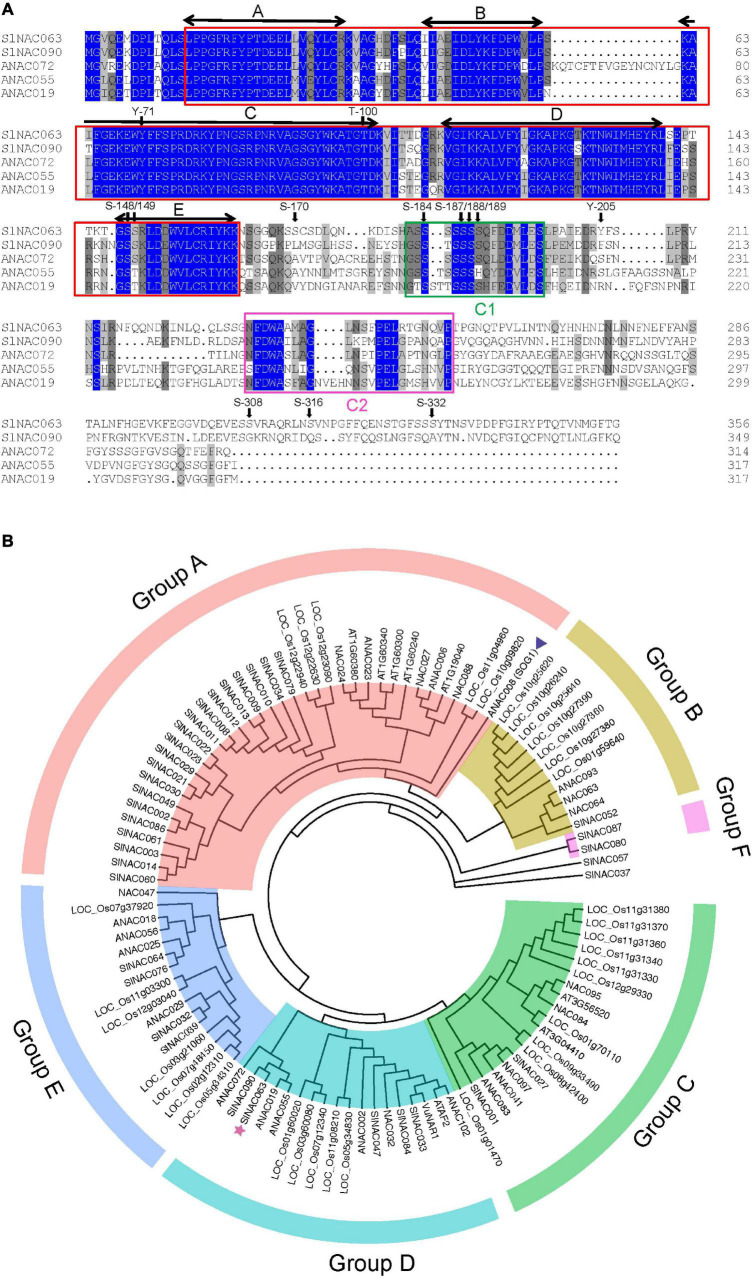
Characterization of SlNAC063 protein. **(A)** Sequence alignment of tomato SlNAC063 with SlNAC090, and *Arabidopsis* ANAC072, ANAC055, and ANAC019 proteins. The conserved NAC domains are indicated by red boxes, with five highly conserved subdomains A–E indicated by two-way arrows. C1 and C2 domains are indicated by green and red boxes, respectively. Predicted phosphorylation sites are indicated by arrows, S, serine; T, threonine; Y, tyrosine. **(B)** Phylogenetic tree analysis of SlNAC063 with other subgroup IIb NAC proteins from tomato, rice, and *Arabidopsis thaliana* and with two previously reported NAC proteins functionally characterized with respect to Al stress, i.e., rice bean VuNAR1 and *Arabidopsis* SUPPRESSOR OF GAMMA RESPONSE 1 (SOG1). Triangle: the position of SOG1. Star: the position of SlNAC063.

The *SlNAC063* gene (Solyc07g063410) encodes a 356-aa protein with a typical NAC domain in its N terminus, which can be divided into five subdomains, namely A, B, C, D, and E (Ooka et al., 2003; Figure 1A). Although NAC proteins have a highly divergent C-terminus, there are two conserved C1 and C2 domains in SlNAC063 and its closely related homologs (Figure 1A).

Tomato SlNAC proteins could be divided into five subfamilies and SlNAC063 belongs to subfamily IIb (Jin et al., 2020). Phylogenetic analysis of this subgroup among tomato, rice, and Arabidopsis showed that these NAC proteins could be further classified into six groups (Figure 1B). Phylogenetically, SlNAC063 and rice bean VuNAR1 are within the same D group, but showed the far distance with Arabidopsis SOG1. Within the group D, SlNAC063 is closely related to SlNAC090 and Arabidopsis ANAC072, ANAC055, and ANAC019 (Figure 1B). Therefore, it is predictable that SlNAC063 might have roles distinct from VuNAR1 and SOG1.

### Expression Pattern of *SlNAC063*

**FIGURE 2:**
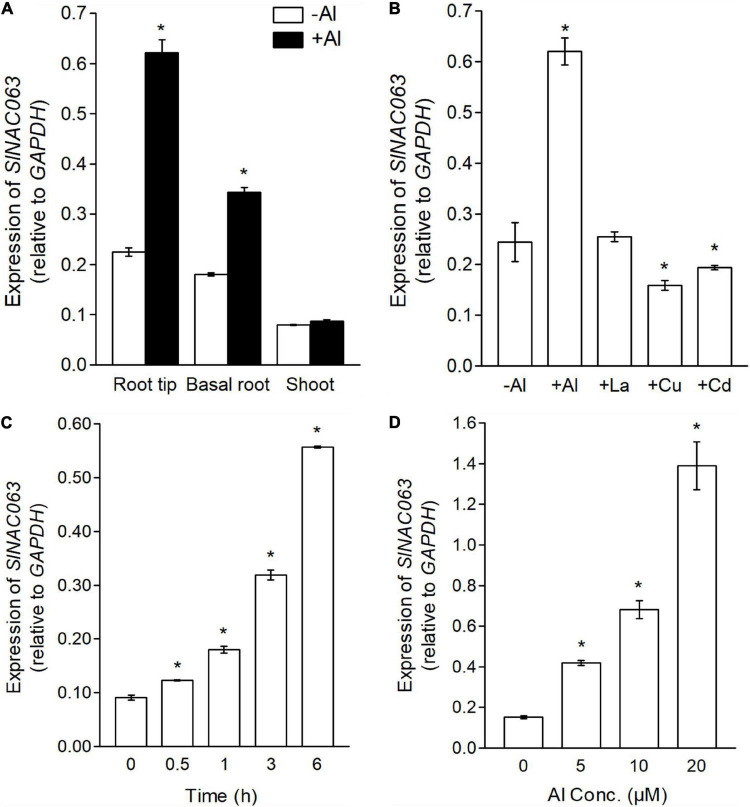
Expression pattern of SlNAC063. **(A)** Analysis of *SlNAC063* expression in different tissues. Seedlings with 3–4 cm root length were transferred to the solution containing 0 or 5 μM Al^3+^ for 6 h. RNA was extracted from root tip (0–1 cm), basal root (1–2 cm), and shoot for expression analysis. **(B)** Expression pattern of *SlNAC063* in response to different metals. Seedlings with 3–4 cm root length were transferred to normal nutrient solution and the solution with 5 μM Al^3+^, 5 μM La^3+^, 1 μM Cu^2+^, and 5 μM Cd^2+^ for 6 h. RNA was extracted from the 0 to 1 cm root tips for expression analysis. **(C)** Time-course expression analysis. Tomato seedlings were treated with 5 μM Al^3+^ for 0, 0.5, 1, 3, and 6 h, respectively, and RNA was extracted from the 0 to 1 cm root tips for expression analysis. **(D)** Al dose-response expression analysis. Tomato seedlings were treated with nutrient solution containing 0, 5, 10, and 20 μM Al^3+^ for 6 h, respectively, and RNA was extracted from the 0 to 1 cm root tips for expression analysis. All treatment solution was 1/5 Hoagland nutrient solution (Pi, 10 μM; pH 5.0). Data are means ± SD (*n* = 3). Glyceraldehyde 3-phosphate dehydrogenase (GAPDH) was used as an internal reference gene; the asterisk indicates significant differences between control and treatment at *P* < 0.05.

In comparison with the shoots, *SlNAC063* mRNA was more abundantly expressed in the roots and the expression was greatly upregulated by Al stress in the roots but not in the shoots (Figure 2A). Spatial expression analysis revealed that Al-induced the expression of *SlNAC063* greater in the root tips (0–1 cm) than in the basal roots (1–2 cm) (Figure 2A). Furthermore, the expression level of *SlNAC063* was not induced by other metals, instead, its expression was slightly repressed by Cd and Cu stress (Figure 2B). A time-dependent expression experiment showed that Al-induced the expression of *SlNAC063* within 30 min of exposure (Figure 2C). In addition, the expression level of *SlNAC063* increased with increasing Al concentrations (Figure 2D).

### SlNAC063 Is a Nucleus-Localized Transcriptional Activator

**FIGURE 3:**
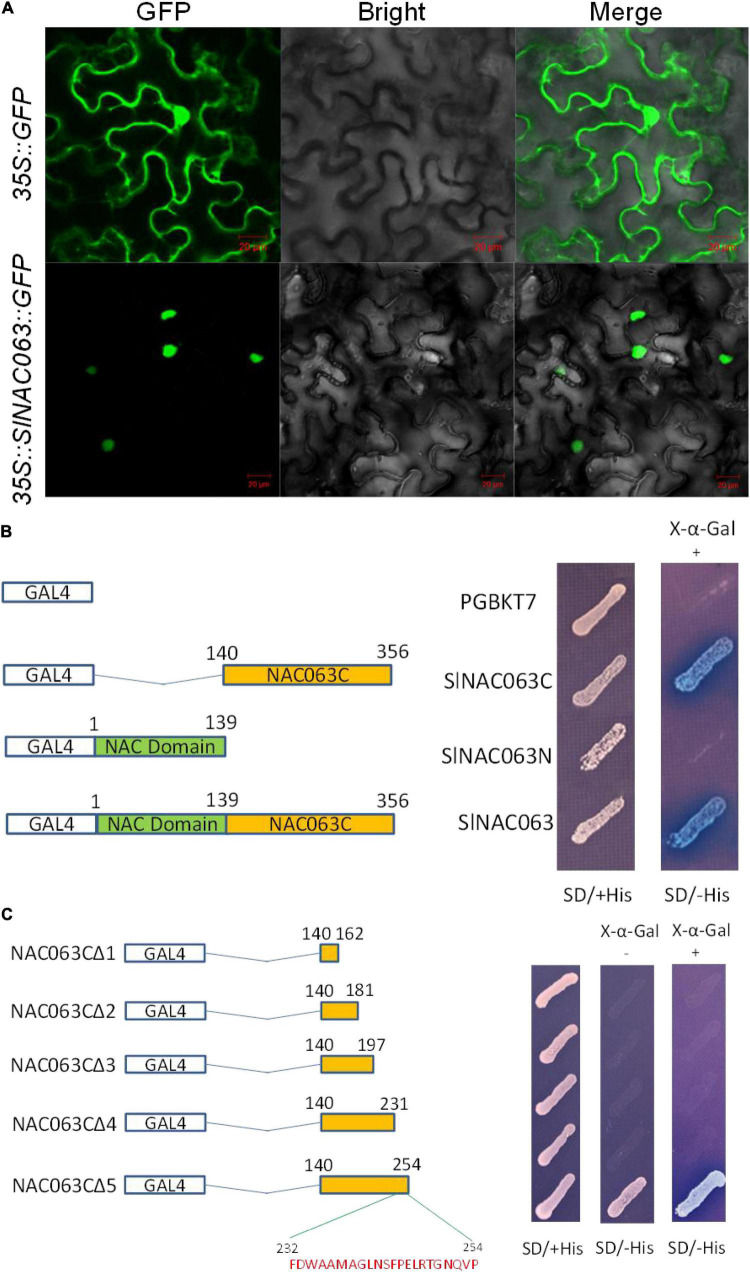
Subcellular localization and transcriptional activation potential of SlNAC063. **(A)** Subcellular localization of SlNAC063. 35S::GFP and 35S::SlNAC063::GFP vectors were, respectively, expressed in tobacco leaves for 48 h, and then GFP signal was observed using a laser confocal microscope. Scale bars = 25 μm. **(B)** The C-terminus of SlNAC063 is responsible for its transcriptional activation activity. The left panel shows the pGBKT7 vectors containing different NAC063 protein fragments or the empty vector. The right panel shows the growth of yeast cells on SD with (+) or without (–) His medium with 4 mM 3-AT and X-α-gal at 30°C for 3 days. The appearance of the blue color was judged as positive results. **(C)** The C2 domain of SlNAC063 C-terminus is critical for its transcriptional activation activity. The left panel shows schematic diagram of the recombined vectors with different SlNAC063 protein fragments. The right panel shows the growth of yeast cells on SD with (+) or without (–) His medium with 4 mM 3-AT and X-α-gal at 30°C for 3 days.

The subcellular localization of SlNAC063 was examined by transiently expressing the *GFP-SlNAC063* fusion gene in tobacco (*Nicotiana benthamiana*) leaf epidermal cells *via* agrobacteria-mediated infiltration. Compared with GFP alone that distributed ubiquitously throughout the cell, SlNAC063-GFP was solely localized to the nucleus (Figure 3A).

To examine whether SlNAC063 has transcriptional activation potential, the full-length SlNAC063 protein (SlNAC063), an N-terminal fragment of SlNAC063 (SlNAC063N), and a C-terminal fragment of SlNAC063 (SlNAC063C) that were fused to the GAL4 DNA-binding domain of the pBGKT7 vector (Figure 3B). Yeast cells carrying with or without three types of SlNAC063 protein grew well on SD medium containing His. However, on SD medium lacking His, only yeast cells carrying SlNAC063 or SlNAC063C grew well and showed β-galactosidase activity in the presence of X-α-Gal. These results indicated that SlNAC063 has transcriptional activation potentials and the C-terminus of SlNAC063 is critical for its transactivation activity.

To further elucidate the sequence motif that confers SlNAC063 transactivation activity, we shortened the SlNAC063C (140–356 aa) from its C-terminal to SlNAC063CΔ5 (140–254 aa), SlNAC063CΔ4 (140–231 aa), SlNAC063CΔ3 (140–197 aa), SlNAC063CΔ2 (140–181 aa), SlNAC063CΔ1 (140–162 aa), and fusion into pBGKT7 vector. The results showed that only SlNAC063CΔ5 grew well as SlNAC063C on SD medium lacking His, but others could not, suggesting that C2 domain (232–254 aa) (FDWAAMAGLNSFPELRTGNQVP) is critical for its transactivation activity (Figure 3C).

### Phenotypic Analysis of SlNAC063 Knockout Lines

To examine the role of SlNAC063 in tomatoes, we generated two independent KO transgenic lines of SlNAC063 using CRISPR/Cas9 technology. Two different mutation types at each CRISPR edited site were found for both transgenic plants (Supplementary Figure 1). The KO line *slnac063*#5 had 4 bp deletion for each sgRNA target, while *slnac063*#7 had 5 bp and 4 bp deletions for sgRNA1 and sgRNA2, respectively. Moreover, no mutations were detected in all other potential off-target sites, suggesting mutagenesis generated at the designed target sites is of high specificity (Supplementary Table 2).

**FIGURE 4:**
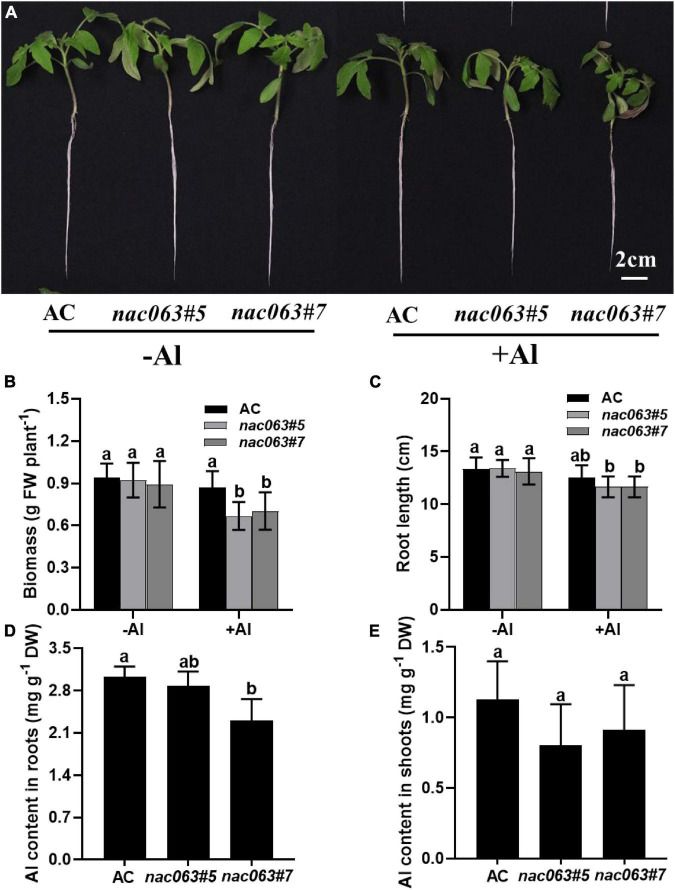
SlNAC063 is positively involved in Al tolerance. **(A)** Phenotypic analysis of slnac063 mutants under Al treatment. **(B,C)** Effect of Al treatment on tomato biomass **(B)** and the longest root length **(C)**. **(D,E)** The aluminum content of tomato roots **(D)** and shoots **(E)**. Seedlings with 3–4 cm root length were transferred to the 1/5 strength Hoagland nutrient solution for acclimation. After 7 days, the acclimatized seedlings were treated with or without 10 μM Al for 7 days. Data are means ± SD [*n* = 10 for **(B,C)**; *n* = 3 for **(D,E)**]. The different letters indicate significant differences (Tukey’s test, *P* < 0.5).

The Al tolerance was compared between wild-type plants and the mutants. Seedlings were subjected to 1/5 Hoagland nutrient solution with 10 μM Pi (pH 5.0) in the presence or absence of 10 μM Al for 1 week. There was no significant difference in biomass between WT plants and two mutants in the absence of Al (Figures 4A,B). While Al stress had no effects on the biomass of WT plants, it significantly reduced that of both mutants. Similarly, although there was no difference in the longest root length between WT plants and two mutants in the absence of Al, root length was significantly reduced by Al stress in both mutants but not in WT plants (Figures 4A,C). Therefore, SlNAC063 is positively involved in Al tolerance in tomatoes.

There are two kinds of Al resistance mechanisms in higher plants. One is external exclusion and the other is internal tolerance. To examine which contributes to SlNAC063-mediated Al tolerance, we compared Al content between WT plants and two mutants in both roots and shoots. Interestingly, lower Al content was observed for the mutants compared with WT plants, especially for *slnac063*#7 (Figure 4D). Accordingly, the shoots of mutants had lower Al content than that of WT plants, though there was no statistical difference (Figure 4E). It appears that internal tolerance mechanisms but not external exclusion mechanisms are involved in SlNAC063-mediated Al tolerance.

### Ribonucleic Acid-Sequencing Analysis of Relatively Long-Term Exposure to Aluminum Stress

To dissect the underlying molecular bases of SlNAC063-mediated Al tolerance, we performed and compared root apex RNA-seq between *slnac063*#5 (*nac063* thereafter) and WT plants after 7 days of treatment with (+Al) or without Al (−Al). As shown in Supplementary Table 3, 12 samples (three independent biological replicates for each treatment) were subjected to RNA-sequencing and produced approximately 6.86 Gb data for each sample. The average genome mapping rate reached 95.58%. With three biological replicates for each treatment, the correlation coefficients showed acceptable reproducibility, indicating the high reliability of our RNA-sequencing data (Supplementary Figure 2).

**FIGURE 5:**
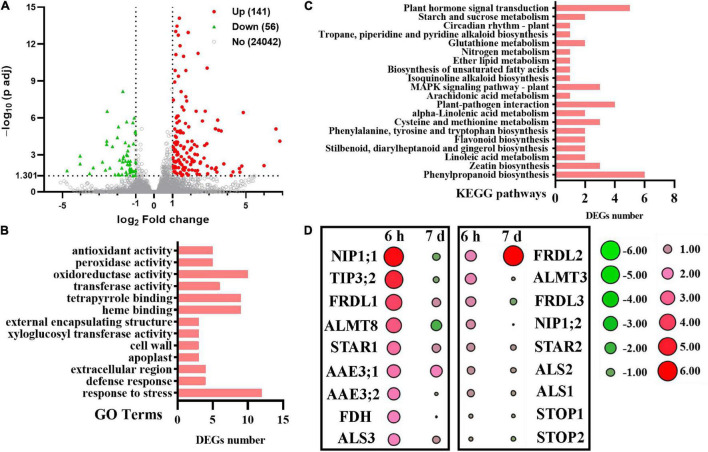
RNA-seq analysis of tomato root tip in response to Al treatment for 7 days. **(A)** Volcano plot of differentially expressed genes (DEGs) in transcriptome (+Al 7 days vs. –Al 7 days). Volcano plot of log2 (fold change) (*x*-axis) vs. –log10 (padj-corrected *P*-value (*y*-axis, representing the probability that the gene is differentially expressed). Red dots denote upregulated DEGs, and green dots denote downregulated DEGs, respectively. Gray dots represent genes with no significant difference. **(B)** The Gene Ontology (GO) biological process classification of DEGs. **(C)** The KEGG pathway classification of DEGs. *X*-axis indicates the number of genes. *Y*-axis indicates the KEGG pathway. **(D)** Heatmaps of the expression profile of Al-tolerance homologous genes in tomato under Al treatment for 6 h and 7 days. The color scale represents fold change (Al vs. CK) of different treatment times.

The DEGs were identified with log2 fold change (FC) ≥ 1 or log2 FC ≤ –1 and with *Q* value (adjusted *P*-value) < 0.05 by DEseq2 (Love et al., 2014). We first analyzed Al-responsive genes in WT plants. After relatively long-term exposure to Al (7 d), we identified 141 upregulated and 56 downregulated genes (Figure 5A and Supplementary Table 4). The Gene Ontology (GO) biological process (BP) analysis showed that these DEGs were quantitatively related to “response to stress,” “oxidoreductase activity,” “tetrapyrrole binding,” and “heme binding” (Figure 5B). The kyoto encyclopedia of genes and genomes (KEGG) pathway analysis showed that most genes were assigned to “phenylpropanoid biosynthesis,” “plant hormone signal transduction,” and “plant-pathogen interaction” (Figure 5C).

When compared the current RNA-seq to a previous one from tomato root apex in response to Al for 6 h where 1620 upregulated and 789 downregulated genes were identified (Jin et al., 2020), a dramatically reduced amount of Al-responsive genes were noticeable. Furthermore, many genes homologous to known Al-tolerance genes dramatically induced during the short-term exposure were found to be attenuated after long-term exposure (Figure 5D). For example, the expression of SlFDH (Solyc02g086880) encoding a formate dehydrogenase induced significantly by 6 h of Al exposure was no longer upregulated after 7 days of exposure (Figure 5D). However, the expression of SlFRDL2 was found to be dramatically induced by long-term exposure of Al (Figure 5D). These results suggest that the molecular mechanisms regulating Al tolerance change with increasing exposure time. In agreement with this suggestion, we have previously demonstrated that two citrate transporter genes were differentially regulated with time in response to Al stress (Liu et al., 2018).

### Genes Affected by SlNAC063 in the Absence of Aluminum Stress

We identified genes whose expression was affected by *SlNAC063* in the absence of Al. There are 1,557 genes upregulated and 636 genes downregulated in *nac063* root apex compared with that of WT plants (Supplementary Figure 3 and Supplementary Table 5). The gene ontology biological process (GOBP) analysis showed that those genes related to “transferase activity,” “tetrapyrrole binding,” “heme binding,” “protein dimerization activity,” and “iron ion binding” are overrepresented among downregulated DEGs. However, upregulated genes were mainly associated with “transcription regulator activity” and “DNA binding transcription activity.” There are many TFs belonging to APETALA2 (AP2), WRKY, NAC, Myb, GAI, RGA, and SCR (GRAS), Heat Shock Factor (HSF), Serum Response Factor (SRF), Helix-Loop-Helix (HLH), and Basic leucine Zipper (bZIP) families whose expression was found to be negatively regulated by SlNAC063 (Supplementary Figures 3D,E). Therefore, it appears that SlNAC063 functions as a negative regulator of downstream TFs.

### Identification of *SlNAC063*-Dependent Aluminum-Responsive Genes

We identified Al-induced genes whose expression was dependent on *SlNAC063*. For Al-induced genes negatively mediated by *SlNAC063*, there are 77 Al-induced genes whose expression could be induced in *nac063* mutants in the absence of Al. However, only two out of them could be further induced by Al in *nac063* mutants, and they are Solyc12g009650 and Solyc03g045140, coding for a hydrophobic seed protein and a probable fatty acid methyltransferase, respectively (Supplementary Figure 4A). For Al-induced genes mediated positively by *SlNAC063*, there are 71 genes whose expression was reduced in *nac063* mutant in the presence of Al stress. However, no gene was found to be repressed in *nac063* both in the presence and absence of Al treatment (Supplementary Figure 4B). Therefore, although *SlNAC063* is able to negatively regulate the expression of many genes in the absence of Al (Figure 5 and Supplementary Figure 3), it is unlikely to function as a transcriptional repressor in Al-induced gene expression.

**FIGURE 6:**
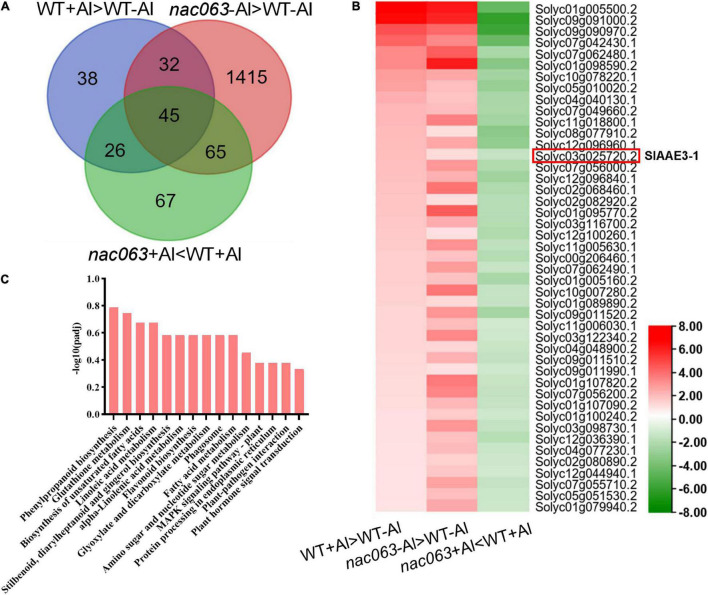
Identification of Al-responsive genes positively regulated by *SlNAC063* under Al stress. **(A)** Venn diagram showing 45 Al-responsive genes whose expression regulation was associated with both Al stress and SlNAC063. **(B)** Expression profiles of 45 potential targeted genes of SlNAC063 *via* heat map analysis. **(C)** The KEGG pathway classification of 45 DEGs.

Next, we identified Al-induced genes whose expression in *nac063* was differentially regulated by Al treatment, i.e., they were upregulated in *nac063* in the absence of Al, but downregulated in *nac063* in the presence of Al. Among 77 Al-induced genes whose expression was upregulated in *nac063* mutant without Al stress, 45 genes were found to be downregulated in *nac063* with Al stress (Figure 6A and Supplementary Table 6). The KEGG pathway analysis showed that these genes were mainly associated with “phenylpropanoid metabolism,” “fatty acid metabolism,” and “dicarboxylate metabolism” (Figure 6B). Notably, a gene encoding oxalyl-CoA synthetase (SlAAE3-1) previously characterized to be implicated in Al tolerance was found to be positively regulated by *SlNAC063* under Al stress (Figure 6C). However, there is no evidence relating other genes with Al tolerance.

**FIGURE 7:**
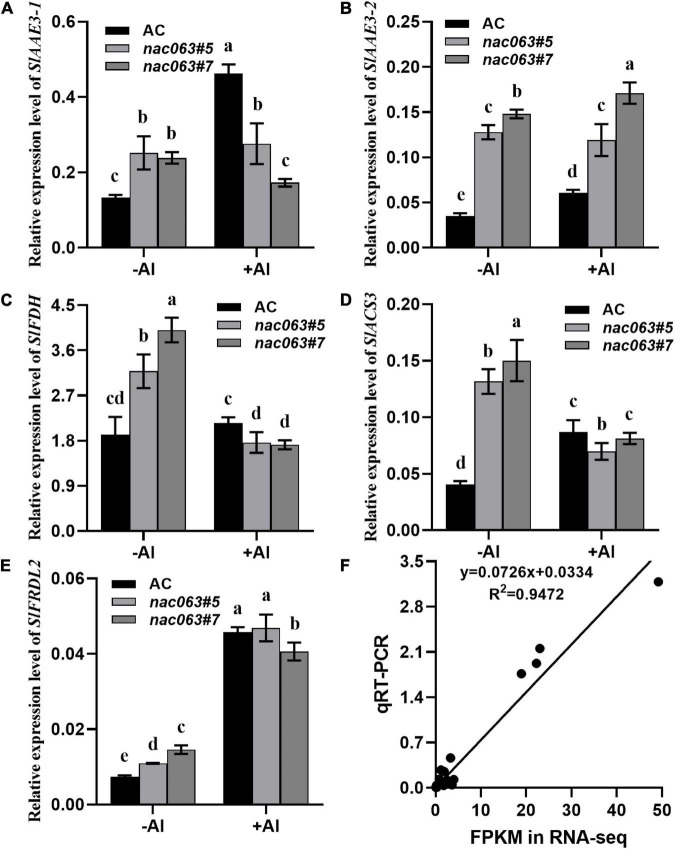
Expression levels of five selected genes in response to Al stress. **(A–E)** RT-qPCR analysis of gene expression of *SlAAE3-1* **(A)**, *SlAAE3-2* **(B)**, *SlFDH* **(C)**, *SlACS3* **(D)**, and *SlFRDL2* **(E)** in AC and *SlNAC063* KO lines under Al stress. *GAPDH* was used as an internal control to normalize expression. Data are means ± SD (*n* = 3 for biological repeats). Different letters indicate significant difference (Tukey’s test, *P* < 0.5). **(F)** Correlation of gene expression levels between RNA-Seq data and real-time quantitative PCR (RT-qPCR) analysis used in **(A–E)** panels.

To confirm the reliability of our RNA-seq data and validate that the expression of *SlAAE3-1* is truly dependent upon *SlNAC063*, RT-qPCR analysis was performed on selected 5 genes between WT plants and two *nac063* mutants. In the absence of Al, the expression of all 5 genes was upregulated in both the *nac063* mutants (Figures 7A–E). While the expression of *SlAAE3-1* and *SlACS3* was induced by Al in WT plants, their induction was repressed in nac063 mutants. No expression difference was found for *SlFDH* and *SlFRDL2* under Al stress between WT plants and *nac063* mutants, suggesting that the expression regulation of both genes in response to Al stress was independent of *SlNAC063*. The expression level of *SlAAE3-2* did not differ between −Al and +Al conditions in *nac063* mutants, suggesting that *SlNAC063* is a master regulator of *SlAAE3-2* expression. A good correlation was observed for their expression between RT-qPCR analysis and RNA-seq (Figure 7F). These results suggest that our RNA-sequencing and gene identification are highly reliable.

## Discussion

The tomato NAC proteins represent a large TF family with 93 members (Jin et al., 2020). Although nearly two dozen of tomato NAC TFs have been functionally characterized with respect to development, and responses to abiotic and biotic stresses, the biological function of most SlNAC genes remains unclear (Jin et al., 2020). Notably, many of these characterized SlNAC TFs belong to the IIb subfamily (Jin et al., 2020; Figure 1). For instance, SlNAC032 (Solyc04g005610) was involved in fruit development (Kou et al., 2016; Ma et al., 2018), and response to stresses (Zhu et al., 2014b; Wang et al., 2016); SlNAC033 (Solyc04g009440) in fruit development (Ma et al., 2014); SlNAC039 (Solyc05g007770) regulates leaf senescence and fruit yield (Ma et al., 2018); SlNAC047 (Solyc06g060230) in chilling tolerance and senescence (Li et al., 2016; Lira et al., 2017); SlNAC064 (Solyc07g063420) in seed development and fruit ripening (Han et al., 2014; Gao et al., 2018); SlNAC076 (Solyc10g006880) in fruit ripening (Kumar et al., 2018); SlNAC084 (Solyc11g017470) positively affects fruit ripening and carotenoid accumulation (Zhu et al., 2014a); SlNAC090 (JA2) (Solyc12g013620) regulates pathogen-induced stomata movement (Du et al., 2014). Previously, SlNAC063 (JA2L) has been shown to be involved in jasmonic acid-mediated stomatal movement during pathogenesis (Du et al., 2014). Here, we demonstrate that SlNAC063 is involved in response to Al stress in tomatoes. This conclusion was drawn based on the following lines of evidence. First, rather than other metals, the expression of *SlNAC063* was specifically induced by Al, which was both time dependent and dose dependent (Figure 2). Second, in comparison with WT plants, CRISPR/Cas9 generated KO lines were more sensitive to Al stress (Figure 4). Finally, the expression of two genes *SlAAE3-1* and *SlAAE3-2* homologous to previously identified Al tolerance gene *VuAAE3* was found to be affected by *SlNAC063* (Figure 7).

We further demonstrate that the internal tolerance mechanism could be implicated in SlNAC063-mediated Al tolerance in tomatoes. Plants employ either an external exclusion mechanism or internal tolerance mechanism to combat with Al stress (Kochian, 1995). In some cases, both mechanisms are evolved by some plants, such as buckwheat, to survive Al toxicity (Ma et al., 1997). Compared to WT plants, *nac063* KO mutants accumulated a slightly lower Al at both the roots and shoots, despite that the mutants were more sensitive to Al stress (Figure 4), ruling out the possibility that SlNAC063 is implicated in external exclusion of Al in tomato roots. Although there are 2213 genes differentially regulated by *SlNAC063* in the absence of Al (Figure 5), only 77 Al-induced genes were found to be affected by *SlNAC063* in the presence of Al (Figure 6A and Supplementary Figure 4). Furthermore, among these genes, few known genes for Al tolerance were present (Supplementary Table 6). Therefore, it appears that SlNAC063 has minor effects on Al tolerance. Nevertheless, it was evident that the expression of *SlAAE3-1* and *SlAAE3-2* was negatively regulated by *SlNAC063* in the absence of Al stress (Figure 6B). It is also notable that Al-induced *SlAAE3-1* requires a functional *SlNAC063* (Figure 7). Given the importance of AAE3-mediated oxalate degradation in response to Al stress (Lou et al., 2016b; Xian et al., 2020; Jin et al., 2021), it is very likely that SlNAC063-mediated Al tolerance in tomato is ascribed to the expression regulation of *SlAAE3-1*.

Yeast one-hybrid experiment suggested that SlNAC063 had transactivation potentials (Figure 3). However, this does not mean that SlNAC063 itself does not have a trans-repressive function against its target gene *in vivo*. In fact, much more genes were found to be upregulated in *nac063* mutants compared with WT plants in the absence of Al and many TFs, including NAC TFs, were found to be negatively regulated by SlNAC063 (Supplementary Figure 3). Intriguingly, we found that the expression regulation of *SlAAE3-1* by *SlNAC063* was dependent on Al stress. At first glance, it seems contradictory that *SlNAC063* differentially regulated *SlAAE3-1* expression in the presence and absence of Al. To our limited knowledge, two possibilities are accountable for such discrepancy. First, SlNAC063 was translationally repressed by Al stress, and the transcriptional induction of *SlNAC063* by Al represents a feedback regulation of gene expression. However, this possibility seems impossible since the expression level of *SlNAC063* was not further induced by Al in *nac063* mutants (Figure 7). Second, novel TF(s) were activated by Al stress to act as a transcriptional activator that coordinates with SlNAC063 to regulate *SlAAE3-1*. A C2H2-type zinc finger TF, STOP1 has been reported to be a master activator of the expression of genes involved in Al tolerance (Iuchi et al., 2007). Recent evidence also showed that the activation of STOP1 protein by Al stress is critical for these downstream gene expressions (Zhang et al., 2019). Therefore, whether STOP1 interacts with SlNAC063 to regulate *SlAAE3-1* expression deserved further investigation. In line with our supposition, it has been reported that the HOX-PBX complex can be converted from a repressor to an activator through differential interactions with other co-regulators, depending on the cellular context (Saleh et al., 2000). A similar scenario has also been reported for Al-induced *AtALMT1* expression in Arabidopsis, where WRKY46 functions as a transcriptional repressor of *AtALMT1* expression despite that Al-induced *AtALMT1* expression was completely abolished in *STOP1* loss-of-function mutants (Zhu et al., 2021).

*In vitro* assay showed that the substrate affinity of the SlAAE3-2 enzyme to oxalate is dramatically lower than that of SlAAE3-1 (data not shown). Moreover, the expression level of *SlAAE3-2* was dramatically lower than that of *SlAAE3-1* (Figure 7). Additionally, Al stress differentially regulates the expression of *SlAAE3-1* and *SlAAE3-2*. The expression of *SlAAE3-2* was independent of Al, whereas that of *SlAAE3-1* was strongly related to Al stress (Figure 7). Therefore, SlAAE3-1 is in a dominant position in regulating oxalate metabolism and plays a major role in regulating Al tolerance, whereas SlAAE3-2 is a functionally minor one. Accumulating evidence showed that oxalate has pivotal roles not only in biological and metabolic processes, but in response to stresses (Franceschi and Nakata, 2005). However, oxalate is also a common constituent of plant-derived human diets. Excess dietary intake of oxalate has the risk of nephrolithiasis (Moe, 2006). Besides, oxalate is also a precursor of β-N-oxalyl-L-α, β-diaminopropionic acid (β-ODAP), a neurotoxin (Xiong et al., 2015; Kumar V. et al., 2016). Therefore, manipulating oxalate metabolism in plants has attracted scientific attention (Kumar et al., 2019). Here, we found that SlNAC063 is an important regulator of *SlAAE3-1* and *SlAAE3-2* expression, thereby providing an alternative to genetically manipulating oxalate metabolism in plants.

## Conclusion

We demonstrate that the tomato NAC TF SlNAC063 is involved in Al tolerance by regulating the expression of *SlAAE3-1*.

## Data Availability Statement

The datasets presented in this study can be found in online repositories. The names of the repository/repositories and accession number(s) can be found in the article/Supplementary Material.

## Author Contributions

WC and JY conceived the study and wrote the manuscript. JJ, HZ, QH, and WC performed the experiments. PL, WF, and JX provided technical assistance. JJ and HZ analyzed the bioinformatic data. All authors contributed to the article and approved the submitted version of the manuscript.

## Conflict of Interest

The authors declare that the research was conducted in the absence of any commercial or financial relationships that could be construed as a potential conflict of interest.

## Publisher’s Note

All claims expressed in this article are solely those of the authors and do not necessarily represent those of their affiliated organizations, or those of the publisher, the editors and the reviewers. Any product that may be evaluated in this article, or claim that may be made by its manufacturer, is not guaranteed or endorsed by the publisher.

## Footnotes

1
https://phytozome-next.jgi.doe.gov/

2
https://www.megasoftware.net/

3
https://services.healthtech.dtu.dk/service.php?NetPhos-3.1

4
http://crispr.hzau.edu.cn/CRISPR2/

5
https://www.ncbi.nlm.nih.gov/bioproject/PRJNA785101/

## References

[B1] Anders S. Huber W. (2010). Differential expression analysis for sequence count data. *Genome Biol.* 11:R106. 10.1186/gb-2010-11-10-r106 20979621 PMC3218662

[B2] Blom N. Gammeltoft S. Brunak S. (1999). Sequence and structure-based prediction of eukaryotic protein phosphorylation sites. *J. Mol. Biol.* 294 1351–1362. 10.1006/jmbi.1999.3310 10600390

[B3] Blom N. Sicheritz-Pontén T. Gupta R. Gammeltoft S. Brunak S. (2004). Prediction of post-translational glycosylation and phosphorylation of proteins from the amino acid sequence. *Proteomics* 4 1633–1649. 10.1002/pmic.200300771 15174133

[B4] Brachmann R. K. Boeke J. D. (1997). Tag games in yeast: the two-hybrid system and beyond. *Curr. Opin. Biotechnol.* 8 561–568. 10.1016/s0958-1669(97)80029-8 9353226

[B5] Chen W. W. Fan W. Lou H. Q. Yang J. L. Zheng S. J. (2017). Regulating cytoplasmic oxalate homeostasis by acyl activating enzyme3 is critical for plant Al tolerance. *Plant Signal. Behav.* 12:e1276688. 10.1080/15592324.2016.1276688 28045586 PMC5289516

[B6] Chen W. W. Tang L. Wang J. Y. Zhu H. H. Jin J. F. Yang J. L. (2022). Research advances in the mutual mechanisms regulating response of plant roots to phosphate deficiency and aluminum toxicity. *Int. J. Mol. Sci.* 23, 1137. 10.3390/ijms23031137 35163057 PMC8835462

[B7] Cheng N. Foster J. Mysore K. S. Wen J. Rao X. Nakata P. A. (2018). Effect of acyl activating enzyme (AAE)3 on the growth and development of *Medicago truncatula*. *Biochem. Biophys. Res. Commun.* 505 255–260. 10.1016/j.bbrc.2018.09.104 30245129

[B8] Cho J. Benoit M. Catoni M. Drost H. G. Brestovitsky A. Oosterbeek M. (2019). Sensitive detection of pre-integration intermediates of long terminal repeat retrotransposons in crop plants. *Nat. Plants* 5 26–33. 10.1038/s41477-018-0320-9 30531940 PMC6366555

[B9] Daspute A. A. Kobayashi Y. Panda S. K. Fakrudin B. Kobayashi Y. Tokizawa M. (2018). Characterization of CcSTOP1; a C2H2-type transcription factor regulates Al tolerance gene in pigeonpea. *Planta* 247 201–214. 10.1007/s00425-017-2777-6 28921050

[B10] Ding Z. J. Yan J. Y. Xu X. Y. Li G. X. Zheng S. J. (2013). WRKY46 functions as a transcriptional repressor of ALMT, regulating aluminum-induced malate secretion in Arabidopsis. *Plant J.* 76 825–835. 10.1111/tpj.12337 24118304

[B11] Du M. M. Zhai Q. Z. Deng L. Li S. Y. Li H. S. Yan L. H. (2014). Closely Related NAC transcription factors of tomato differentially regulate stomatal closure and reopening during pathogen attack. *Plant Cell* 26 3167–3184. 10.1105/tpc.114.128272 25005917 PMC4145139

[B12] Fan W. Lou H. Q. Gong Y. L. Liu M. Y. Cao M. J. Liu Y. (2015). Characterization of an inducible C2H2-type zinc finger transcription factor VuSTOP1 in rice bean (Vigna umbellata) reveals differential regulation between low pH and aluminum tolerance mechanisms. *New Phytol.* 208 456–468. 10.1111/nph.13456 25970766

[B13] Fan W. Lou H. Q. Gong Y. L. Liu M. Y. Wang Z. Q. Yang J. L. (2014). Identification of early Al-responsive genes in rice bean (*Vigna umbellata*) roots provides new clues to molecular mechanisms of Al toxicity and tolerance. *Plant Cell Environ.* 37 1586–1597. 10.1111/pce.12258 24372448

[B14] Foster J. Kim H. U. Nakata P. A. Browse J. (2012). A previously unknown oxalyl-CoA synthetase is important for oxalate catabolism in Arabidopsis. *Plant Cell* 24 1217–1229. 10.1105/tpc.112.096032 22447686 PMC3336115

[B15] Foster J. Nakata P. A. (2014). An oxalyl-CoA synthetase is important for oxalate metabolism in Saccharomyces cerevisiae. *FEBS Lett.* 588 160–166. 10.1016/j.febslet.2013.11.026 24291261

[B16] Franceschi V. R. Nakata P. A. (2005). Calcium oxalate in plants: formation and function. *Annu. Rev. Plant Biol.* 56 41–71. 10.1146/annurev.arplant.56.032604.144106 15862089

[B17] Gao Y. Wei W. Zhao X. D. Tan X. L. Fan Z. Q. Zhang Y. P. (2018). A NAC transcription factor, NOR-like1, is a new positive regulator of tomato fruit ripening. *Hortic. Res.* 5:75. 10.1038/s41438-018-0111-5 30588320 PMC6303401

[B18] Gao Z. X. Dong B. Y. Cao H. Y. He H. Yang Q. Meng D. (2020). Time series RNA-seq in pigeonpea revealed the core genes in metabolic pathways under aluminum stress. *Genes* 11:380. 10.3390/genes11040380 32244575 PMC7230159

[B19] Garcia-Oliveira A. L. Benito C. Prieto P. Menezes R. D. A. Rodrigues-Pousada C. Guedes-Pinto H. (2013). Molecular characterization of TaSTOP1 homoeologues and their response to aluminium and proton (H^+^) toxicity in bread wheat (*Triticum aestivum* L.). *BMC Plant Biol* 13:134. 10.1186/1471-2229-13-134 24034075 PMC3848728

[B20] Goodstein D. M. Shu S. Q. Howson R. Neupane R. Hayes R. D. Fazo J. (2012). Phytozome: a comparative platform for green plant genomics. *Nucleic Acids Res.* 40 1178–1186. 10.1093/nar/gkr944 22110026 PMC3245001

[B21] Gupta S. Van Eck J. (2016). Modification of plant regeneration medium decreases the time for recovery of *Solanum lycopersicum* cultivar M82 stable transgenic lines. *Plant Cell Tissue Organ. Cult.* 127 417–423. 10.1007/s11240-016-1063-9

[B22] Han Q. Q. Song Y. Z. Zhang J. Y. Liu L. F. (2014). Studies on the role of the SlNAC3 gene in regulating seed development in tomato (*Solanum lycopersicum*). *J. Hortic. Sci. Biotechnol.* 89 423–429. 10.1080/14620316.2014.11513101

[B23] Huang S. Gao J. You J. F. Liang Y. Guan K. Yan S. (2018). Identification of STOP1-like proteins associated with aluminum tolerance in sweet sorghum (*Sorghum bicolor* L.). *Front. Plant Sci.* 9:258. 10.3389/fpls.2018.00258 29541086 PMC5835670

[B24] Iuchi S. Koyama H. Iuchi A. Kobayashi Y. Kitabayashi S. Ikka T. (2007). Zinc finger protein STOP1 is critical for proton tolerance in Arabidopsis and coregulates a key gene in aluminum tolerance. *Proc. Natl. Acad. Sci. USA* 104 9900–9905. 10.1073/pnas.0700117104 17535918 PMC1887543

[B25] Jin J. F. He Q. Y. Li P. F. Lou H. Q. Chen W. W. Yang J. L. (2021). Genome-wide identification and gene expression analysis of acyl-activating enzymes superfamily in tomato (*Solanum lycopersicum*) under aluminum stress. *Front. Plant Sci.* 12:754147. 10.3389/fpls.2021.754147 34925406 PMC8674732

[B26] Jin J. F. Wang Z. Q. He Q. Y. Wang J. Y. Li P. F. Xu J. M. (2020). Genome-wide identification and expression analysis of the NAC transcription factor family in tomato (*Solanum lycopersicum*) during aluminum stress. *BMC Genomics* 21:288. 10.1186/s12864-020-6689-7 32264854 PMC7140551

[B27] Kochian L. V. (1995). Cellular mechanisms of aluminum toxicity and resistance in plants. *Annu. Rev. Plant Physiol. Plant Mol. Biol.* 46 237–260. 10.1146/annurev.pp.46.060195.001321

[B28] Kochian L. V. Piñeros M. A. Liu J. P. Magalhaes J. V. (2015). Plant adaptation to acid soils: the molecular basis for crop aluminum resistance. *Annu. Rev. Plant Biol.* 66 571–598. 10.1146/annurev-arplant-043014-114822 25621514

[B29] Kou X. H. Liu C. Han L. H. Wang S. Xue Z. H. (2016). NAC transcription factors play an important role in ethylene biosynthesis, reception and signaling of tomato fruit ripening. *Mol. Genet. Genomics* 291 1205–1217. 10.1007/s00438-016-1177-0 26852223

[B30] Kumar R. Tamboli V. Sharma R. Sreelakshmi Y. (2018). NAC-NOR mutations in tomato Penjar accessions attenuate multiple metabolic processes and prolong the fruit shelf life. *Food Chem.* 259 234–244. 10.1016/j.foodchem.2018.03.135 29680049

[B31] Kumar S. Stecher G. Tamura K. (2016). MEGA7: molecular evolutionary genetics analysis version 7.0 for bigger datasets. *Mol. Biol. Evol.* 33 1870–1874. 10.1093/molbev/msw054 27004904 PMC8210823

[B32] Kumar V. Chattopadhyay A. Ghosh S. Irfan M. Chakraborty N. Chakraborty S. (2016). Improving nutritional quality and fungal tolerance in soya bean and grass pea by expressing an oxalate decarboxylase. *Plant Biotechnol. J.* 14 1394–1405. 10.1111/pbi.12503 26798990 PMC11389089

[B33] Kumar V. Irfan M. Datta A. (2019). Manipulation of oxalate metabolism in plants for improving food quality and production. *Phytochemistry* 158 103–109. 10.1016/j.phytochem.2018.10.029 30500595

[B34] Kundu A. Das S. Basu S. Kobayashi Y. Koyama H. Ganesan M. (2019). GhSTOP1, a C2H2 type zinc finger transcription factor is essential for Aluminum and proton stress tolerance and lateral root initiation in cotton. *Plant Biol.* 21 35–44. 10.1111/plb.12895 30098101

[B35] Li G. Z. Wang Z. Q. Yokosho K. Ding B. Fan W. Gong Q. Q. (2018). Transcription factor WRKY22 promotes aluminum tolerance via activation of OsFRDL4 expression and enhancement of citrate secretion in rice (Oryza sativa). *New Phytol.* 219 149–162. 10.1111/nph.15143 29658118

[B36] Li X. D. Zhuang K. Y. Liu Z. M. Yang D. Y. Ma N. N. Meng Q. W. (2016). Overexpression of a novel NAC-type tomato transcription factor, SlNAM1, enhances the chilling stress tolerance of transgenic tobacco. *J. Plant Physiol.* 204 54–65. 10.1016/j.jplph.2016.06.024 27518221

[B37] Lira B. S. Gramegna G. Trench B. A. Alves F. R. R. Silva E. M. Silva G. F. F. (2017). Manipulation of a Senescence-Associated Gene Improves Fleshy Fruit Yield. *Plant Physiol.* 175 77–91. 10.1104/pp.17.00452 28710129 PMC5580748

[B38] Liu H. Ding Y. D. Zhou Y. Q. Jin W. Q. Xie K. B. Chen L. L. (2017). CRISPR-P 2.0: An Improved CRISPR-Cas9 Tool for Genome Editing in Plants. *Mol. Plant* 10 530–532. 10.1016/j.molp.2017.01.003 28089950

[B39] Liu J. Pineros M. A. Kochian L. V. (2014). The role of aluminum sensing and signaling in plant aluminum resistance. *J. Integr. Plant Biol.* 56 221–230. 10.1111/jipb.12162 24417891

[B40] Liu M. Y. Lou H. Q. Chen W. W. Piñeros M. A. Xu J. M. Fan W. (2018). Two citrate transporters co-ordinately regulate citrate secretion from rice bean root tip under aluminum stress. *Plant Cell Environ.* 41 809–822. 10.1111/pce.13150 29346835

[B41] Lou H. Q. Fan W. Jin J. F. Xu J. M. Chen W. W. Yang J. L. (2020). A NAC-type transcription factor confers aluminium resistance by regulating cell wall-associated receptor kinase 1 and cell wall pectin. *Plant Cell Environ.* 43 463–478. 10.1111/pce.13676 31713247

[B42] Lou H. Q. Gong Y. L. Fan W. Xu J. M. Liu Y. Cao M. J. (2016a). A formate dehydrogenase confers tolerance to aluminum and low pH. *Plant Physiol.* 171 294–305. 10.1104/pp.16.01105 27021188 PMC4854670

[B43] Lou H. Q. Fan W. Xu J. M. Gong Y. L. Jin J. F. Chen W. W. (2016b). An oxalyl-CoA synthetase is involved in oxalate degradation and aluminum tolerance. *Plant Physiol.* 172 1679–1690. 10.1104/pp.16.01106 27650448 PMC5100784

[B44] Louvet O. Doignon F. Crouzet M. (1997). Stable DNA-binding yeast vector allowing high-bait expression for use in the two-hybrid system. *Biotechniques* 23 816–820. 10.2144/97235bm11 9383543

[B45] Love M. I. Huber W. Anders S. (2014). Moderated estimation of fold change and dispersion for RNA-seq data with DESeq2. *Genome Biol.* 15:550. 10.1186/s13059-014-0550-8 25516281 PMC4302049

[B46] Ma J. F. Ryan P. R. Delhaize E. (2001). Aluminium tolerance in plants and the complexing role of organic acids. *Trends Plant Sci.* 6 273–278. 10.1016/s1360-1385(01)01961-6 11378470

[B47] Ma J. F. Zheng S. J. Matsumoto H. Hiradate S. (1997). Detoxifying aluminium with buckwheat. *Nature* 390 569–570.9403684

[B48] Ma N. N. Feng H. L. Meng X. Li D. Yang D. Y. Wu C. G. (2014). Overexpression of tomato SlNAC1 transcription factor alters fruit pigmentation and softening. *BMC Plant Biol.* 14:351. 10.1186/s12870-014-0351-y 25491370 PMC4272553

[B49] Ma X. M. Zhang Y. J. Turečková V. Xue G. P. Fernie A. R. Mueller-Roeber B. (2018). The NAC Transcription Factor SlNAP2 Regulates Leaf Senescence and Fruit Yield in Tomato. *Plant Physiol.* 177 1286–1302. 10.1104/pp.18.00292 29760199 PMC6052983

[B50] Maher W. Forster S. Krikowa F. Snitch P. Chapple G. Craig P. (2001). Measurement of trace elements and phosphorus in marine animal and plant tissues by low-volume microwave digestion and ICP-MS. *Atom. Spectrosc.* 22 361–370.

[B51] McCormick S. Niedermeyer J. Fry J. Barnason A. Horsch R. Fraley R. (1986). Leaf disc transformation of cultivated tomato (L. esculentum) using *Agrobacterium tumefaciens*. *Plant Cell Rep.* 5 81–84. 10.1007/BF00269239 24248039

[B52] Moe O. W. (2006). Kidney stones: pathophysiology and medical management. *Lancet* 367 333–344. 10.1016/S0140-6736(06)68071-9 16443041

[B53] Moreno-Alvarado M. García-Morales S. Trejo-Téllez L. I. Hidalgo-Contreras J. V. Olsen A. N. Ernst H. A. (2017). Aluminum Enhances Growth and Sugar concentration, alters macronutrient status and regulates the Expression of NAC Transcription Factors in Rice. *Trends Plant Sci.* 8:73. 10.3389/fpls.2017.00073 28261224 PMC5306397

[B54] Ohyama Y. Ito H. Kobayashi Y. Ikka T. Morita A. Kobayashi M. (2013). Characterization of *AtSTOP1* orthologous genes in tobacco and other plant species. *Plant Physiol.* 162 193–196. 10.1104/pp.113.218958 23749850 PMC3729772

[B55] Olsen A. N. Ernst H. A. Leggio L. L. Skriver K. (2005). NAC transcription factors: structurally distinct, functionally diverse. *Trends Plant Sci.* 10 79–87. 10.1016/j.tplants.2004.12.010 15708345

[B56] Ooka H. Satoh K. Doi K. Nagata T. Otomo Y. Murakami K. (2003). Comprehensive analysis of NAC family genes in Oryza sativa and Arabidopsis thaliana. *DNA Res.* 10 239–247. 10.1093/dnares/10.6.239 15029955

[B57] Peng C. Liang X. Liu E. E. Zhang J. J. Peng X. X. (2017). The oxalyl-CoA synthetase-regulated oxalate and its distinct effects on resistance to bacterial blight and aluminium toxicity in rice. *Plant Biol.* 19 345–353. 10.1111/plb.12542 28039904

[B58] Saleh M. Rambaldi I. Yang X. Featherstone M. S. (2000). Cell signalling switches HOX-PBX complexes from repressors to activators of transcription mediated by histone deacetylases and histone acetyltransferase. *Mol. Cell. Biol.* 20 8623–8633. 10.1128/MCB.20.22.8623-8633.2000 11046157 PMC102167

[B59] Sawaki Y. Iuchi S. Kobayashi Y. Kobayashi Y. Ikka T. Sakurai N. (2009). STOP1 regulates multiple genes that protect Arabidopsis from proton and aluminum toxicities. *Plant Physiol.* 150 281–294. 10.1104/pp.108.134700 19321711 PMC2675709

[B60] Sawaki Y. Kobayashi Y. Kihara-Doi T. Nishikubo N. Kawazu T. Kobayashi M. (2014). Identification of a STOP1-like protein in Eucalyptus that regulates transcription of Al tolerance genes. *Plant Sci.* 223 8–15. 10.1016/j.plantsci.2014.02.011 24767110

[B61] Sjogren C. A. Bolaris S. C. Larsen P. B. (2015). Aluminum-dependent terminal differentiation of the Arabidopsis root tip is mediated through an ATR-. ALT2-, and SOG1-regulated transcriptional response. *Plant Cell* 27 2501–2515. 10.1105/tpc.15.00172 26320227 PMC4815104

[B62] Tokizawa M. Kobayashi Y. Saito T. Kobayashi M. Iuchi S. Nomoto M. (2015). Sensitive to proton rhizotoxicity1, calmodulin binding transcription activator2, and other transcription factors are involved in aluminum-activated malate transporter1 expression. *Plant Physiol.* 167 991–1003. 10.1104/pp.114.256552 25627216 PMC4348791

[B63] Wang G. D. Zhang S. Ma X. C. Wang Y. Kong F. Y. Meng Q. W. (2016). A stress-associated NAC transcription factor (SlNAC35) from tomato plays a positive role in biotic and abiotic stresses. *Physiol. Plant.* 158 45–64. 10.1111/ppl.12444 26991441

[B64] Wang Z. P. Xing H. L. Dong L. Zhang H. Y. Han C. Y. Wang X. C. (2015). Egg cell-specific promoter-controlled CRISPR/Cas9 efficiently generates homozygous mutants for multiple target genes in Arabidopsis in a single generation. *Genome Biol.* 16:144. 10.1186/s13059-015-0715-0 26193878 PMC4507317

[B65] Wu W. Lin Y. Chen Q. Peng W. Peng J. Tian J. (2018). Functional conservation and divergence of soybean GmSTOP1 members in proton and aluminum tolerance. *Front. Plant Sci.* 9:570. 10.3389/fpls.2018.00570 29755502 PMC5932199

[B66] Xian P. Q. Cai Z. D. Cheng Y. B. Lin R. B. Lian T. X. Ma Q. B. (2020). Wild soybean oxalyl-CoA synthetase degrades oxalate and affects the tolerance to cadmium and aluminum stresses. *Int. J. Mol. Sci.* 21:8869. 10.3390/ijms21228869 33238600 PMC7700444

[B67] Xiong J. L. Xiong Y. C. Bai X. Kong H. Y. Tan R. Y. Zhu H. (2015). Genotypic variation in the concentration of β-N-oxalyl-L-α, β-diaminopropionic acid (β-ODAP) in grass pea (*Lathyrus sativus* L.) seeds is associated with an accumulation of leaf and pod β-ODAP during vegetative and reproductive stages at three levels of water stress. *J. Agric. Food Chem.* 63 6133–6141. 10.1021/acs.jafc.5b01729 26027639

[B68] Xu J. M. Fan W. Jin J. F. Lou H. Q. Chen W. W. Yang J. L. (2017). Transcriptome Analysis of Al-Induced Genes in Buckwheat (*Fagopyrum esculentum* Moench) Root apex: new insight into al toxicity and resistance mechanisms in an Al Accumulating Species. *Front. Plant Sci.* 8:1141. 10.3389/fpls.2017.01141 28702047 PMC5487443

[B69] Yang J. L. Fan W. Zheng S. J. (2019). Mechanisms and regulation of aluminum-induced secretion of organic acid anions from plant roots. *J. Zhejiang Univ. Sci. B* 20 513–527. 10.1631/jzus.b1900188 31090277 PMC6568218

[B70] Zhang H. Y. Wang X. H. Dong L. Wang Z. P. Liu B. Lv J. (2017). MISSA 2.0: an updated synthetic biology toolbox for assembly of orthogonal CRISPR/Cas systems. *Sci. Rep.* 7:41993. 10.1038/srep41993 28155921 PMC5290471

[B71] Zhang Y. Zhang J. Guo J. L. Zhou F. L. Singh S. Xu X. (2019). F-box protein RAE1 regulates the stability of the aluminum-resistance transcription factor STOP1 in Arabidopsis. *Proc. Natl. Acad. Sci. USA* 116 319–327. 10.1073/pnas.1814426116 30559192 PMC6320511

[B72] Zhao H. Huang W. Zhang Y. Zhang Z. Li Y. Tang C. (2018). Natural variation of CsSTOP1 in tea plant (*Camellia sinensis*) related to aluminum tolerance. *Plant Soil* 431 71–87. 10.1007/s11104-018-3746-y

[B73] Zhu M. K. Chen G. P. Zhou S. Tu Y. Wang Y. Dong T. T. (2014a). A New Tomato NAC (NAM/ATAF1/2/CUC2) Transcription Factor, SlNAC4, Functions as a positive regulator of fruit ripening and carotenoid accumulation. *Plant Cell Physiol.* 55 119–135. 10.1093/pcp/pct162 24265273

[B74] Zhu M. K. Hu Z. L. Zhou S. Wang L. L. Dong T. T. Pan Y. (2014b). Molecular characterization of six tissue-specific or stress-inducible genes of NAC Transcription Factor Family in Tomato (*Solanum lycopersicum*). *J. Plant Growth Regul.* 33 730–744. 10.1007/s00344-014-9420-6

[B75] Zhu X. Wang P. Bia Z. Herde M. Ma Y. Li N. (2021). Calmodulin-like protein CML24 interacts with CAMTA2 and WRKY46 to regulate ALMT1-dependent Al resistance in Arabidopsis thaliana Calmodulin-like protein CML24 interacts with CAMTA2 and WRKY46 to regulate ALMT1-dependent Al resistance in Arabidopsis thaliana. *New Phytol.* 2021:17812. 10.1111/nph.17812 34665465

