# Supplementary material for: The Tomato Transcription Factor SlNAC063 Is Required for Aluminum Tolerance by Regulating SlAAE3-1 Expression
Source: Front Plant Sci. 2022 Mar 15;13:826954. doi: 10.3389/fpls.2022.826954 (PMC8965521; doi:10.3389/fpls.2022.826954)
Supplement: Supplementary file 1 [file Data_Sheet_1.docx]

**Supplementary Figure S1**

**Supplementary Figure S1.** Identification of two independent CRISPR/Cas9 mutation lines of *NAC063*. **(A)** Schematic diagram of two *NAC063* genomic target sites. Two target sites were set at the third exon of *NAC063*, red triangles indicate target sites. **(B)** Gene editing analysis of *nac063#5* and *nac063#7* mutants. Red bases indicate target sequences, black lines indicate PAMs, and short blue lines indicate missing bases.
