# Supplementary material for: The Tomato Transcription Factor SlNAC063 Is Required for Aluminum Tolerance by Regulating SlAAE3-1 Expression
Source: Front Plant Sci. 2022 Mar 15;13:826954. doi: 10.3389/fpls.2022.826954 (PMC8965521; doi:10.3389/fpls.2022.826954)
Supplement: Supplementary file 2 [file Data_Sheet_2.docx]

## Supplementary Figure 2


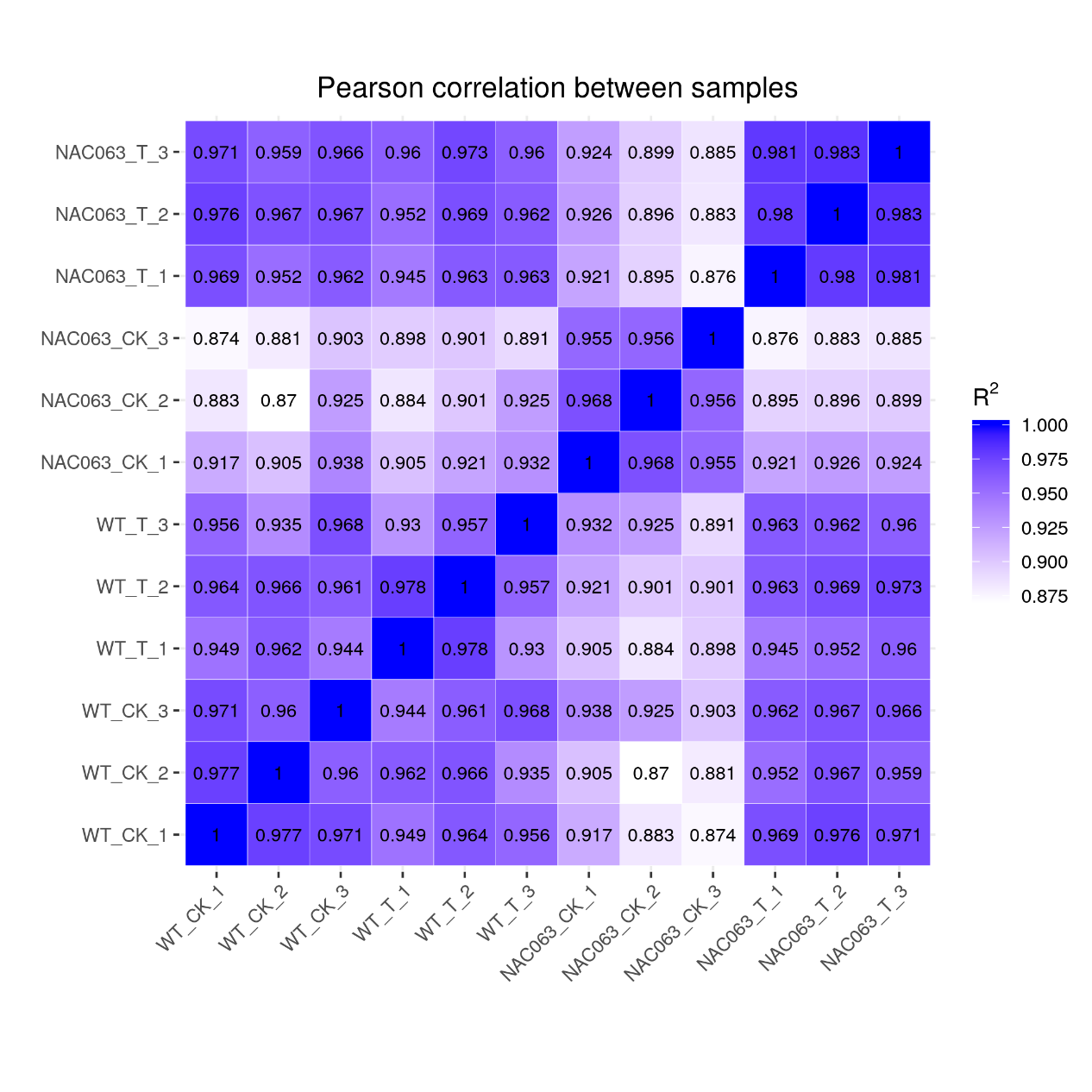


**Supplementary Figure 2.** The correlations of RNA-sequencing biological replicates of WT and *nac063* mutant.
