# Supplementary material for: The Tomato Transcription Factor SlNAC063 Is Required for Aluminum Tolerance by Regulating SlAAE3-1 Expression
Source: Front Plant Sci. 2022 Mar 15;13:826954. doi: 10.3389/fpls.2022.826954 (PMC8965521; doi:10.3389/fpls.2022.826954)
Supplement: Supplementary file 3 [file Data_Sheet_3.docx]

**Supplementary Figure S3**

**Supplementary Figure S3.** Identification of SlNAC063-regulated genes in the absence of Al stress in tomato root tip. **(A)** Volcano plot of differentially expressed genes (DEGs) in transcriptome (AC-Al 7d and *slnac063*-Al 7d). **(B)** The classification of the down-regulated DEGs according to GO biological process. **(C)** The classification of the up-regulated DEGs according to GO biological process. **(D)** The class and the number of up-regulated transcription factor (TF) genes. **(E)** Heatmap of expression profiles of up-regulated NAC family genes in *nac063* mutants.
