# Supplementary material for: The Tomato Transcription Factor SlNAC063 Is Required for Aluminum Tolerance by Regulating SlAAE3-1 Expression
Source: Front Plant Sci. 2022 Mar 15;13:826954. doi: 10.3389/fpls.2022.826954 (PMC8965521; doi:10.3389/fpls.2022.826954)
Supplement: Supplementary file 4 [file Data_Sheet_4.docx]

## Supplementary Figure 4

**Supplementary Figure 4.** Venn diagrams of Al-responsive genes whose expression was regulated by SlNAC063 either negatively (A) or positively (B). It is worth noting that only 2 genes were found to be negatively regulated by *SlNAC063* and no gene was found to be positively regulated by *SlNAC063* under Al stress.
